# Pretreatment with myo-inositol in non polycystic ovary syndrome patients undergoing multiple follicular stimulation for IVF: a pilot study

**DOI:** 10.1186/1477-7827-10-52

**Published:** 2012-07-23

**Authors:** Franco Lisi, Piero Carfagna, Mario Montanino Oliva, Rocco Rago, Rosella Lisi, Roberta Poverini, Claudio Manna, Elena Vaquero, Donatella Caserta, Valeria Raparelli, Roberto Marci, Massimo Moscarini

**Affiliations:** 1 Center for Reproductive Medicine Research, Clinica Villa Mafalda, Rome, Italy; 2 GENESIS Center for Reproductive Medicine, Rome, Italy; 3 Department of Surgery, Tor Vergata - University of Rome, Rome, Italy; 4 Department of Women Health and Territorial Medicine, Sapienza - University of Rome, Sant’Andrea Hospital, Rome, Italy; 5 Department of Internal Medicine and Medical Specialties, Sapienza - University of Rome, Rome, Italy; 6 Department of Biomedical Sciences and Advanced Therapies, Section of Obstetrics and Gynecology, University Hospital of Ferrara, Ferrara, Italy

**Keywords:** Myo-inositol, Inositol, Follicle, Stimulation, IVF, ICSI, Oocytes, Embryos

## Abstract

**Background:**

Aim of this pilot study is to examine the effects of myo-inositol administration on ovarian response and oocytes and embryos quality in non PolyCystic Ovary Syndrome (PCOS) patients undergoing multiple follicular stimulation and *in vitro* insemination by conventional *in vitro* fertilization or by intracytoplasmic sperm injection.

**Methods:**

One hundred non-PCOS women aged <40 years and with basal FSH <10 mUI/ml were down-regulated with triptorelin acetate from the mid-luteal phase for 2 weeks, before starting the stimulation protocol for oocytes recovery. All patients received rFSH, at a starting dose of 150 IU for 6 days. The dose was subsequently adjusted according to individual response. Group B (n = 50) received myo-inositol and folic acid for 3 months before the stimulation period and then during the stimulation itself. Group A (n-50) received only folic acid as additional treatment in the 3 months before and through treatment.

**Results:**

Total length of the stimulation was similar between the two groups. Nevertheless, total amount of gonadotropins used to reach follicular maturation was found significantly lower in group B. In addition, the number of oocytes retrieved was significantly reduced in the group pretreated with myo-inositol. Clinical pregnancy and implantation rate were not significantly different in the two groups.

**Conclusions:**

Our findings suggest that the addition of myo-inositol to folic acid in non PCOS-patients undergoing multiple follicular stimulation for in-vitro fertilization may reduce the numbers of mature oocytes and the dosage of rFSH whilst maintaining clinical pregnancy rate. Further, a trend in favor of increased incidence of implantation in the group pretreated with myo-inositol was apparent in this study. Further investigations are warranted to clarify this pharmacological approach, and the benefit it may hold for patients.

## Background

Myo-inositol is an isomer of a C6 sugar alcohol that belongs to the vitamin B complex group [1]. Some studies suggested that myo-inositol could play an important role in cellular morphogenesis and cytogenesis, in the synthesis of lipids, in the creation of cell membranes and in cell growth [2,3]. It is also a precursor of phospholipids, which are responsible for the generation of important intracellular signals in mammalian oocytes and in the resumption of meiotic maturation [4-6]. The presence of myo-inositol in human body fluids and its effect on the *in vitro* maturation of oocytes in rats have led some authors to state that myo-inositol concentration in follicular fluid is significantly higher in follicles containing good quality oocytes than in follicles containing poor quality oocytes [7].

Myo-inositol also regulates, via signal transduction pathways, the secretion of some exocrine glands such as pancreas and other organs, including the ovaries. In the oocytes these intracellular pathways are involved in the release of cortical granules, in the inhibition of polyspermy, in the completion of meiosis and in the activation of the cell cycle that subsequently results in embryonic development [8]. It has been hypothesized that intrafollicular myo-inositol concentration and oocyte quality might be connected because inositol phospholipids (of which myo-inositol is a precursor) are held responsible for important intracellular signals essential for oocyte development, and because myo-inositol itself seems to improve oocytes *in vitro* maturation [8,9].

Recently, the role of myo-inositol has powerfully emerged in the pathogenesis of polycystic ovary syndrome (PCOS), in particular linked with insulin resistance. In fact, some of the actions of insulin are mediated by putative inositol-containing phosphoglycan (IPG) mediators, also known as putative insulin mediators or second messengers. These mediators are generated by hydrolysis of glycosylphosphatidylinositol lipids and/or proteinated species located in the outer leaflet of the cell membrane. Two different IPG have been identified: (i) the d-chiro-IPG mediator, which activates pyruvate dehydrogenase phosphatase, and (ii) the MYO-IPG which inhibits cyclic AMP-dependent protein kinase [10,11]. A positive role of myo-inositol in insulin-resistant women with PCOS could depend on defects in the insulin IPG-mediated signaling pathway, that seems to be primarily implicated in the pathogenesis of insulin resistance in this clinical setting [12,13]. Accordingly, myo-inositol has been classified as an insulin sensitizing agent and it is commonly used in PCOS treatment [14-16]. By rescuing the ovarian response to endogenous gonadotropins, myo-inositol reduces hyperandrogenemia and re-establishes menstrual cyclicity and ovulation, increasing the chance of a spontaneous pregnancy [17,18]. Its use in human is safe and only the highest dose (12 g/day) induced mild gastrointestinal side effects such as nausea, flatus and diarrhea [19].

The aim of this paper was to illustrate the progress recently made in the use of myo-inositol in fertility treatment and in particular to discuss its effects on ovarian response and oocyte quality in non-PCOS patients undergoing multiple follicular stimulations and *in vitro* inseminations by conventional *in vitro* fertilization (IVF) or by intracytoplasmic sperm injection (ICSI).

## Methods

**Figure 1:**
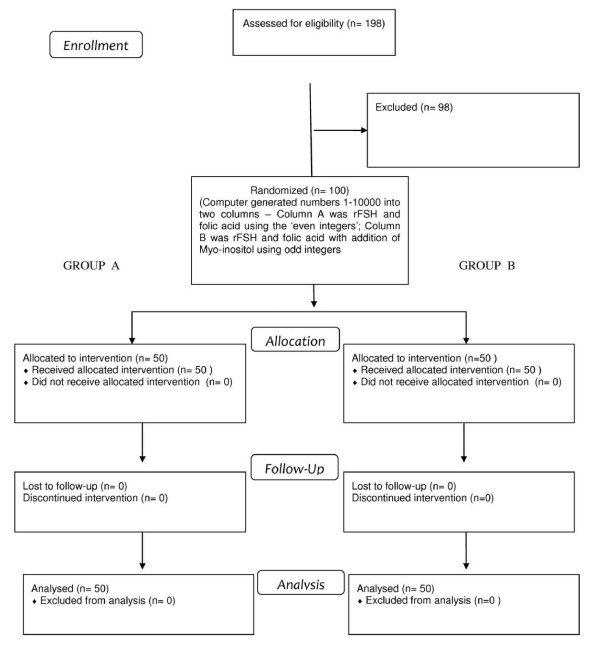
Flow chart showing the progress through the phases of the trial

This prospective, randomized, open-label, multicenter pilot study compared patients treated with 400 μg of folic acid for the 3 months before and during rFSH administration, following the long protocol (Group A, n = 50) with patients that received a daily dose of 4,000 mg of myo-inositol into two administrations/day in addition to 400 μg of folic acid for the 3 months before and during rFSH administration, following the long protocol (Group B, n = 50). The trial adhered to the Helsinki Declaration and the protocol was approved by the Institutional Review Boards. All patients signed a written informed consent before entering the study. Inclusion criteria were age < 40 years old and basal FSH level <10 mUI/ml. Patients presenting diagnostic criteria for PCOS [14] or other concomitant endocrine and metabolic diseases such as hypothyroidism, hyperthyroidism, diabetes mellitus, androgen-secreting tumors, adrenal hyperplasia, Cushing's syndrome, hyper-prolactinemia, and all patients that underwent hormonal treatment in the previous 3 months were excluded from the study. Day 2 FSH, LH, 17β-estradiol (E2) and prolactin (PRL) levels were measured in the previous 6 months. Patients' BMI was between 18 and 28 and obese women (BMI greater than 30 kg/m^2^) were excluded from the study: Six patients of the initial cohort had a BMI greater than 30 kg/m2 (6/198; 3%). All patients began treatment during a set period —January 2011 to January 2012 (“treatment run”)— and were allocated to the treatment groups using block randomization in a computer generated sequence: computer generated numbers 1-10000 into two columns – Column A was rFSH and folic acid using the ‘even integers’; Column B was rFSH and folic acid with addition of myo-inositol using odd integers (Figure 1). Allocation to group A or group B was decided on the day of first consultation and was not known to doctors who performed the monitoring of follicular development, changes in the amount of daily International Units of rFSH, egg retrieval, *in vitro* fertilization, embryo transfer and decided luteal support.

Patients enrolled in the study were recommended ICSI or IVF after evaluation of their partner's semen. Patients were down-regulated with triptorelin acetate (0.1 mg, SC; Ferring, Italy) from the mid-luteal phase (day 21 of the previous cycle) for 2 weeks, before starting the stimulation protocol for oocyte recovery. In all patients, ovarian suppression was confirmed by ultrasound scan (absence of ovarian activity, ovarian cyst formation and endometrial proliferation) and serum dosage of E2 levels (≤40 pg/ml) before starting exogenous gonadotropins administration. If ovarian suppression was not met, down-regulation was extended for a further week. Group A received folic acid at a dose of 400 μg per day for 3 months before and during treatment with gonadotropins and received rFSH (Gonal F, Merck) at a starting dose of 150 IU for 6 days. The dose was subsequently adjusted according to individual response. Group B received the same gonadotropins treatment and the stimulation was preceded by the administration of 2,000 mg of myo-inositol twice a day and 400 μg per day of folic acid for 3 months before starting follicular stimulation and continued during the stimulation itself. When conventional criteria for human chorionic gonadotropin (hCG) administration were met (at least three follicles with a mean diameter >17 mm), recombinant human hCG, (250 μg SC; Ovitrelle, Merck Serono) was given (at least 24 hours after the last rFSH administration) to induce final oocyte maturation. Transvaginal oocyte retrieval was scheduled 35-37 hours after the trigger injection. IVF or ICSI, depending on semen parameters (data not shown), were then performed. Oocytes retrieval, IVF or ICSI and embryos transfer were carried out according to our usual clinical practice [20]. Embryo quality was assessed morphologically, 2 days after fertilization using a grading system [21]. Grade 1 and 2 embryos have no or very few fragments in the cytoplasm with equal size blastomeres and therefore are considered the best embryos. Grade 3 and 4 embryos have significant or severe fragmentation; little cytoplasmic fragmentation with blastomeres of distinctively unequal size [21]. The luteal phase was supported by 800 mg/day transvaginal-micronized progesterone (Progeffik, Effik Italia S.p.A, Milan, Italy) and treatment was continued until either a serum pregnancy test result was negative or an embryonic heart beat was sonographically confirmed. Serum hCG level was measured 14 days after oocyte retrieval. A slight and transitory increase in β-hCG level was defined as a biochemical pregnancy. A gestational sac with fetal heartbeat movement seen on transvaginal ultrasound scan 4 weeks after embryo transfer, confirmed clinical pregnancy.

### Statistical analysis

The number of treated patients and controls was computed with respect to a two-tailed Student *t* test for independent groups, considering a (i) difference in gonadotropins dosage required to reach follicular maturation to be detected between patients and controls |δ| ≥ 15%, (ii) type I error probability α = 0.05 and power 1 − β = 0.72; this resulted in n = 50 for group. Sample size calculation was performed using the software nQuery Advisor, version 5.0 (Statistical Solutions, Saugus, Massachusetts). Analysis was performed according to an intention to treat principle. Student’s *t*-test for independent samples was used to evaluate statistical differences between groups for continuous variables. Comparisons between proportions were conducted using the Pearson’s chi-square test.

Data are presented as mean ± SD. Probability values < 0.05 were regarded as statistically significant. All calculations were made with the computer programme STATISTICA 7 (StatSoft, Tulsa, OK, USA).

## Results and discussion

Patients' age was similar in the two groups as well as BMI, FSH, LH and E2 basal levels. After the down-regulation treatment, LH level was significantly higher in patients pre-treated with myo-inositol [Group A: 1.6 ± 0.9 mUI/ml; Group B: 2.7 ± 1.1mUI/ml, p < 0.01], while basal FSH and basal E2 were similar (Table 1). The total length of the stimulation was similar between the two groups [Group A: 11.7 ± 1.8 days; Group B: 11.8 ± 1.5 days; p = 0.64]. The total amount of gonadotropins used to reach follicular maturation was significantly reduced in group B [Group A: 2,479 ± 979 IU; Group B: 2,084 ± 648 IU; p <0.05]. E2 peak level on the day of hCG administration was found to be lower, but not statistically significant, in group A [Group A: 1.312 ± 629; Group B 1.516 ± 942 pg/ml, p = 0.12] and the number of oocytes per patient retrieved resulted significantly higher in group A [Group A: 7.6 ± 3.8 per patient (Tot. 380); Group B: 5,9 ± 2,4 oocytes per patient (Tot. 297), p < 0.01] (Table 2). In addition the number (mean ± SD) of Metaphase II eggs, number of inseminated eggs, number of 2PN oocytes and number of embryos were significantly higher in group A compared with group B (Table 2). Fertilization rate, cleavage rate, percentage of grade I and II embryos, number of patients receiving embryos and number of embryos received were similar in both groups (Table 2).

**Table 1:** Baseline and stimulation characteristics of patients in group without (Group A) and with (Group B) pretreatment with Myo-inositol

	**No myo-inositol**	**Yes myo-inositol**	**p**
	**Group A**	**Group B**	
	**N = 50**	**N = 50**	
*No. of patients*	50	50	
*Age (year)*	33.3 ± 2.8	34.4 ± 3.4	0.09
*BMI*	22.9 ± 3.3	22.7 ± 2.6	0.88
*FSH (mIU/ml), basal*	7.3 ± 1.5	7.2 ± 2	0.35
*LH (mIU/ml), basal*	4.7 ± 2	4.9 ± 2	0.87
*17β-estradiol (E2) (pg/ml),*	47.2 ± 17.8	43.6 ± 12.7	0.20
*basal*			
*FSH (mIU/ml), down*	4.7 ± 1.5	4.6 ± 2.2	0.87
*regulation*			
*LH (mIU/ml), down*	1.6 ± 0.9	2.7 ± 1.1	<0.01
*regulation*			
*17β-estradiol (E2) (pg/ml),*	26.4 ± 20	24.4 ± 14.5	0.31
*down regulation*			
*rFSH treatment days*	11.7 ± 1.8	11.8 ± 1.5	0.64
*Total rFSH dose (IU)*	2,479 ± 979	2,084 ± 648	<0.05
*E2 (pg/ml), day of hCG*	1,312 ± 629	1,516 ± 640	0.12
*Total length of stimulation*	11.7 ±1.8	11.8 ± 1.5	0.64

**Table 2:** Study outcomes in the two treatment groups

	**No myo-inositol**	**Yes myo-inositol**	**p**
	**Group A**	**Group B**	
	**N = 50**	**N = 50**	
*No. oocytes retrieved/patient*	7.6 ± 3.8	5.9 ± 2.4	<0.01
*Mean ± SD*			
*No. metaphase II/patient*	6.3 ± 2.9	4.8 ± 2.2	<0.05
*Mean ± SD*			
*No. inseminated eggs/patient*	6 ± 2.7	4.8 ± 2.2 (240)	<0.05
*Mean ± SD*			
*No. 2PN oocytes/patient*	4.3 ± 2.3	3.3 ± 1.8/163	<0.01
*Mean ± SD*			
*Fertilization rate (2PN/inseminated oocytes)*	70.8 ± 20.4	68.4 ± 19.2	0.7
*(%) ± SD*			
*No. Embryos/patient*	3.58 ± 2.1	2.5 ± 1 .1	<0.001
*Mean ± SD*			
*Cleavage rate (embryos/2PN oocytes)*	82.2 ± 29.9	84.5 ± 28.7	0.7
*(%) ± SD*			
*No. embryos Grade I and II : number*	173 (96.6%)	117 (93.6%)	0.2
*and percentage of total*			
*No. of patients receiving*	1	47 (94%)	1
*embryos (%)*			
*No. of embryos transferred per*	2.4 ± 1	2.2 ± 0.8	0.39
*starting patients Mean ± SD*			
*No. Clinical pregnancies*	12/47 (25.5%)	14/47 (29.8%)	0.52
*No. of fetal hearts*	16 (13.3%)	21 (18.7%)	0.08
*(implantation rate)*			

The number of clinical pregnancies was also similar [Group A: 12/47 (25,5%); Group B: 14/47 (29,8%), p = 0.52]. Finally, even the implantation rate (number of gestational sac with fetal heartbeat/total number of embryos transferred) was similar in the two groups of patients [Group A: 13,3%; Group B: 18,7%, p = 0.08] (Table 2).

PCOS affects 5%-10% of women in reproductive age, and is the most common cause of infertility due to anovulation. Insulin resistance is common in PCOS women, regardless of their body mass index. The importance of insulin resistance in PCOS is also suggested by the fact that insulin-sensitizing compounds have been proposed as putative treatments to solve hyperinsulinemia-induced dysfunction of ovarian response to endogenous gonadotropins. Rescuing ovarian response to endogenous gonadotropins reduces hyperandrogenemia and re-establishes menstrual cyclicity and ovulation, increasing the chance of a spontaneous pregnancy. Among insulin-sensitizing compounds, myo-inositol has been shown to be able to restore spontaneous ovarian activity, and consequently fertility, in most patients with PCOS [18,22,23]. Myo-inositol may be a useful tool in the treatment of PCOS patients undergoing ovulation induction for its insulin-sensitizing activity. Myo-inositol has also been proposed as an adjuvant in multiple follicular stimulation for IVF in patients known to suffer from PCOS : the pretreatment with myo-inositol and folic acid was shown to reduce germinal vesicles and degenerated oocytes at ovum pick-up, without compromising the total number of retrieved oocytes [8,24].

However, the intent of this study was to assess the role of myo-inositol in cellular morphogenesis and cytogenesis. At present not many data are available regarding the action and effects of myo-inositol in non-PCOS women in childbearing age, in spontaneous ovulation and in stimulation cycles. Increasing evidence supports the physiological and therapeutic role of myo-inositol in human reproduction and in particularly in oogenesis playing an important role in cell morphogenesis and cytogenesis, lipid synthesis, structure of cell membranes and cell growth [2,3]. Some studies have shown that myo-inositol is incorporated into phosphoinositides and inositol phosphates in rabbit embryos [25] and can enhance bovine blastocyst development from *in vitro* culture with medium supplemented with myo-inositol [26]. Results from these studies support the notion that myo-inositol serves as a precursor for the synthesis of phosphoinositides. This constitutes the phosphatidylinositol (PtdIns) signal transduction system known to be involved in the regulation of diverse cellular functions including cell proliferation [27]. During ovulation induction for an IVF cycle two important parameters need to be monitored: E2 concentration and follicles size/number. An increase in these two factors has been correlated with a higher level of myo-inositol in the follicular fluid [7]. In 1992 Chiu and Tam [28] demonstrated that serum myo-inositol could be a trophic factor responsible for promoting *in vitro* development of preimplantation embryos.

Myo-inositol is an important element of the follicular microenvironment that plays a crucial role in oocyte maturation. In fact, in Assisted Reproduction, supplementation of myo-inositol is positively related to oocyte meiotic progression of germinal vesicles in rats, increasing the intracellular calcium oscillations [4,28,29]. In patients treated with exogenous gonadotropins plus myo-inositol there was a significant reduction in the number of oocytes retrieved and in the number of follicles recruited. For this reason we can assume that this approach could be adopted to reduce the risk of hyperstimulation. Overall, these results provide a further support to the hypothesis that myo-inositol may promote the meiotic maturation acting on intracellular signal transduction in calcium pathways [29-31]. A complete meiotic maturation requires intracellular changes associated with both nuclear and cytoplasmic components [30]. At present, although most of the morphological and biochemical changes during maturation are well documented, a complete identification of specific factors that directs these changes is lacking [32]. The fertilizability of oocytes, their ability to initiate embryo splitting, and the subsequent preimplantation development are now considered a fundamental part of a proper assessment of cytoplasmic maturation [33]. It is interesting to highlight how LH level during down regulation was higher in the group pretreated with myo-inositol [Group A: 1.6 ± 0,9 mIU/ml; Group B: 2.7 ± 1.1 mIU/ml, p < 0.01]. Although the meaning of this finding is not clear, we can speculate that the increased rate of circulating LH is responsible for the higher level of E2 on the day of HCG administration (although not significant in our group of patients) and for the lower number of follicles recruited, suggesting that it may be a cofactor for better oocytes and embryos quality [34].

## Conclusion

The addition of myo-inositol seems to reduce gonadotropin dosage and the number of MII oocytes retrieved in non-PCOS patients pretreated with myo-inositol for 3 months. However, this study is underpowered to evaluate IVF outcomes like implantation and clinical pregnancy with the mechanism of improved oocyte competence. Therefore a subsequent adequately powered RCT is underway.

## Competing interests

The authors declare that they have no competing interests.

## Authors' contributions

All the authors participated in designing the study, patients' enrollment, analysis of results and preparation of manuscript. FL, PC, MMO, RR, RL, RP, CM, EV, DC, RM, MM participated in designing, recruiting and treating patients, in the analysis of results and the preparation of the manuscript. VR participated in the analysis of data and in the preparation of manuscript. All authors read and approved the final version of the manuscript.
